# Co-occurrence of acute hemolytic anemia and methemoglobinemia in a 74-year-old female with G6PD deficiency: A case report

**DOI:** 10.1097/MD.0000000000042826

**Published:** 2025-06-13

**Authors:** Binze Chen, Yanxia Han

**Affiliations:** a Department of Clinical Laboratory, Gansu Provincial Maternity and Child-Care Hospital, Lanzhou, Gansu, China; b Department of Clinical Laboratory, The 940th Hospital of Joint Logistics Support Force of Chinese People’s Liberation Army, Lanzhou, Gansu, China.

**Keywords:** case report, favism, G6PD deficiency, hemolytic anemia, methemoglobinemia

## Abstract

**Rationale:**

Acute hemolysis and methemoglobinemia rarely coexist in patients with glucose-6-phosphate dehydrogenase (G6PD)-deficient disease. Methylene blue is widely recognized as the first-line treatment for methemoglobinemia; however, it can worsen hemolysis owing to its oxidative effects in patients with G6PD deficiency.

**Patient concerns:**

A rare case of acute hemolytic anemia and methemoglobinemia induced by ingestion of fresh raw fava beans in a 74-year-old female with G6PD deficiency was reported in this paper.

**Diagnoses:**

Laboratory test results revealed anemia (low hemoglobin level of 81 g/L) with evidence of hemolysis (high reticulocyte count 11.93%, high indirect bilirubin 155.7 μmol/L, high proportion of poikilocytes [16.5%] observed in peripheral blood smears). Arterial blood gas analysis showed a high methemoglobin level (7.3%). Detection of mutations in G6PD confirmed the diagnosis of G6PD deficiency.

**Interventions:**

The administration of methylene blue exacerbated the hemolysis of this patient, resulting in a sustained decrease in hemoglobin levels from 81 to 38 g/L. Methylene blue treatment was replaced by vitamin C and methylprednisolone upon diagnosis of G6PD deficiency.

**Outcomes:**

The clinical symptoms of this patient were gradually improved after vitamin C and methylprednisolone treatment, and 1 month after discharge, the hemoglobin level recovered to within the normal range.

**Lessons:**

Our case highlights the importance of evaluating G6PD deficiency as soon as the patient is first diagnosed with methemoglobinemia. Although acute hemolytic anemia and methemoglobinemia rarely co-occur in elderly patients with G6PD deficiency, care should be taken with methylene blue, which can worsen hemolysis. The fava bean plays an important role in the hemolytic crisis. Therefore, it is crucial to identify the cause of oxidative stress and its co-occurrence to prevent further oxidative stress-induced hemolysis.

## 1. Introduction

Glucose-6-phosphate dehydrogenase (G6PD) deficiency is a common X-linked hereditary hemolytic disease affecting approximately 500 million people worldwide.^[1]^ They are usually asymptomatic unless exposed to oxidative stress conditions such as infections, certain drugs, or ingestion of fava beans. As a common cause of hemolytic anemia in G6PD-deficient patients, favism shows different clinical features in different age groups after ingestion of fava beans.^[2]^ Vicine and convicine are the 2 components of fava beans that inducing a hemolytic crisis during favism.^[2]^

Additionally, fava bean ingestion can also lead to methemoglobinemia, which is rarely congenital, but is primarily acquired through exposure to oxidants. Methemoglobin (MetHgb) is an abnormally oxidized form of hemoglobin (Hgb) in which the ferrous (Fe2+) iron in heme is oxidized to the ferric (Fe3+) state.^[3]^

Acute hemolysis and methemoglobinemia rarely coexist in patients with G6PD-deficient disease. Methylene blue is widely recognized as the first-line treatment for methemoglobinemia; however, it can worsen hemolysis owing to its oxidative effects in patients with G6PD deficiency.^[3]^ Therefore, obtaining a detailed medical history from patients at risk of hemolysis is crucial for effective disease management, particularly among those with G6PD deficiency that has not been previously diagnosed.

Globally, the ingestion of fava beans remains the primary cause of acute hemolytic crisis associated with G6PD deficiency in all age group,^[2]^ although there have been limited reports concerning elderly populations. Herein, this case reported a 74-year-old female patient who presented with severe hemolytic anemia and methemoglobinemia due to the ingestion of fresh fava beans.

## 2. Case report/case presentation

A 74-year-old female was admitted to the emergency department because of yellow sclerae and skin without obvious inducement, accompanied by asthenia, anorexia, nausea, and red urine. Her medical history revealed hypertension and type-2 diabetes mellitus, with no recent infections or new medications. She denied any history of food or drug allergies.

Upon examination in the emergency department, her heart rate was 83 beats per minute, and respiratory rate was 22 breaths per minute; however, her blood pressure was 176/73 mm Hg. Jaundice without fever was observed during physical examination, while the rest of the examination yielded unremarkable findings. Laboratory test results (Table 1) indicated low Hgb and red blood cell (RBC) levels and elevated indirect bilirubin and lactate dehydrogenase (LDH) levels. Urinalysis revealed hyperbilirubinuria. Following admission, supplemental oxygen therapy was initiated along with other treatments, including rehydration, dexamethasone for anti-inflammatory effects, and cefuroxime ester sodium for anti-infection effects. Based on arterial blood gas analysis results showing a high MetHb level of 7.3% (Table 1), a provisional diagnosis of methemoglobinemia was made, and the patient received intravenous administration of methylene blue at a dose of 50 mg (1–2 mg/kg) in the emergency department setting. Subsequent follow-up examinations revealed normal chest X-ray findings, and electrocardiogram readings were unremarkable. Blood cell analysis revealed an increased proportion of reticulocytes, whereas the proportion of poikilocytes was elevated in peripheral blood smears.

**Table 1:** Laboratory test results of the patient.

Parameters	Result	Reference range	Parameters	Result	Reference range
WBC (×10^9^/L)	12.90	4.0–10.0	FIB (g/L)	4.37	2.00–4.00
Neutrophils (×10^9^/L)	9.64	2.0–7.0	D-Dimer (µg/L FEU)	7501	0–700
Lymphocytes (×10^9^/L)	2.43	0.8–4.0	Urine occult blood test (mg/L)	+++(7.5)	–
RBC (×10^12^/L)	2.35	3.68–5.13	Urobilinogen (μmol/L)	+(34)	–
Hgb (g/L)	81	113–151	MetHgb (%)	7.3	0–1.5
PLT (×10^9^/L)	168	101–320	Reticulocyte (%)	11.93	0.5–1.5
TB (µmol/L)	172.0	0.0–21.0	Red cell debris (%)	1.0	–
IB (µmol/L)	155.7	3.0–14.0	Proportion of poikilocyte (%)	16.5	–
LDH (U/L)	991	120–250	Folate (ng/mL)	13.3	3.1–20.5
UREA (mmol/L)	9.78	2.60–7.50	Vitamin B12 (pg/mL)	701.0	187.0–883.0
CREA (µmol/L)	78	41–81	Ferritin (ng/mL)	578.86	7.0–323.0
CRP (mg/L)	52.71	0.00–8.00	HIV	Negative	Negative
PCT (ng/mL)	0.67	0.00–0.50	Coombs test	Negative	Negative

CREA = creatinine, CRP = c-reactive protein, FIB = fibrinogen, HIV = human immunodeficiency virus, Hgb = hemoglobin, IB = indirect bilirubin, LDH = lactate dehydrogenase, MetHgb = methemoglobin, PCT = procalcitonin, PLT = platelet, RBC = red blood cells, TB = total bilirubin, WBC = white blood cell.

**Figure 1.**
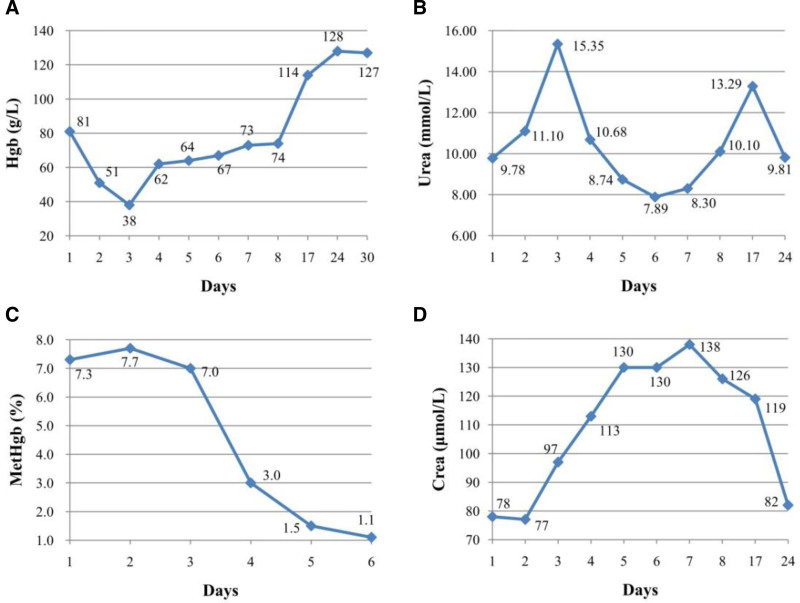
Trend of different blood parameters of the patient throughout the hospital stay and discharge: (A) Hgb, (B) urea, (C) MetHgb, (D) CREA. Day 1: admission, Days 2 to 3: treatment with methylene blue, Days 4 to 8: treatment with washed RBCs, Days 17 to 30: discharge until recovery. CREA = creatinine, Hgb = hemoglobin, MetHgb = methemoglobin.

On the second day after admission, the patient’s Hgb level dropped further to 38 g/L. Unexpectedly, the patient’s renal function deteriorated with an increase in urea and creatinine concentrations, resulting in acute renal failure. Following treatment with 2 units of washed RBCs and fluid therapy, the patient’s Hgb level increased to 62 g/L (Fig. 1). Since the patient’s hemolytic anemia continued to worsen, a detailed history was obtained to determine the cause of hemolysis. Two days before symptom onset, the patient picked and consumed fresh raw fava beans. According to the patient’s recent ingestion of fresh raw fava beans and laboratory test results suggestive of acute hemolysis, G6PD deficiency was suspected and subsequently confirmed by the detection of a missense mutation in exon 12 of the G6PD gene. Treatment with Vitamin C (5 mL/piece) and methylprednisolone (40 mg/bottle) was initiated, while methylene blue was discontinued due to confirmation of favism. Follow-up laboratory tests (RBC 2.22 × 10^12^/L; Hgb 74 g/L, WBC 8.51 × 10^9^/L; no significant abnormalities in other indicators) revealed gradual improvement in clinical symptoms prior to discharge. Subsequently, the patient was discharged and advised to avoid fava beans and drugs that could trigger hemolysis as well as weekly checkups until normality resumed. One month after discharge, the patient’s Hgb level recovered to within the normal range of 127 g/L.

## 3. Discussion

G6PD is a crucial erythrocyte enzyme that plays a vital role in preventing cellular damage from reactive oxidants by catalyzing the first reaction of the pentose phosphate pathway, generating nicotinamide adenine dinucleotide phosphate (NADPH) and ribose necessary for DNA synthesis.^[4]^ Patients with G6PD deficiency cannot generate sufficient NADPH to protect themselves against oxidative stress, which leads to acute intravascular hemolysis. Most people are often asymptomatic unless exposed to oxidative stress conditions, such as infections, certain drugs, or ingestion fava beans. In this case, the trigger event was ingestion of fresh raw fava beans, and the patient exhibited acute hemolysis.

Enzyme activity measurement may miss individuals with normal phenotypes but positive genotypes, especially female heterozygous patients.^[5]^ As seen in our case, the DNA sequencing result of the 74-year-old female patient showed a missense mutation in exon 12 of G6PD; thus, the patient was diagnosed with favism. She had never experienced such symptoms in the past, but only this time did, she experience acute hemolysis after ingestion of fresh raw fava beans. The late onset of favism in this patient was suspected to be attributed to decreased enzyme activity in the elderly, given the fact that G6PD activity gradually decreases with age in G6PD-deficient red cells.^[1]^ Interestingly, G6PD-deficient individuals do not develop into favism every time after ingestion of fava beans. Raw fava beans are more likely to induce hemolytic crisis than cooked, frozen, or canned beans,^[2]^ suggesting that the type of fava beans may play an important role in the hemolytic crisis. Therefore, picking and eating fresh raw fava beans may be the main reason for hemolysis, since the patient had never consumed fresh raw fava beans before.

To our best knowledge, few reports exist on elderly patients and only 25% adults that with G6PD deficiency manifest a hemolytic crisis after the ingestion of fava beans.^[2]^ They present different degrees of severity attacked by favism due to G6PD deficiency, and the age of onset plays an important role in clinical presentation; once severe and life-threatening hemolysis occurs, patients require hospitalization and blood transfusion.^[2,6–8]^ In our case, the 74-year-old female patient had never experienced such symptoms before but developed severe renal failure only at this time. Following treatment with 2 units of washed RBCs and fluid therapy, the patient’s Hgb level increased to 62 g/L, which takes approximately 3 to 4 weeks to return to normal (Fig. 1). Although G6PD has a good overall prognosis, attention must be paid to acute renal failure as a significant complication arising from severe hemolysis, potentially leading to acute tubular necrosis.^[9]^

Several oxidizing agents that induce hemolysis may also trigger methemoglobinemia in patients with G6PD deficiency,^[9]^ which can reduce oxygen transport levels under physiological conditions and increase the risk of tissue hypoxia.^[10]^ The decrease in NADPH in G6PD-deficient patients can lead to oxidative stress in the body, resulting in elevated MetHgb levels.^[11]^ Methemoglobinemia is typically treated with an intravenous infusion of methylene blue (1–2 mg/Kg) over a period of 5-30 mins.^[12]^ Methylene blue can be reduced to leukomethylene blue, which enhances the ability of erythrocytes to reduce MetHgb,^[13]^ it also acts as an oxidant and may further increase oxidative stress.^[14]^ According to the literature, higher doses of methylene blue have been associated with hemolytic anemia independent of MetHg.^[12]^ As observed in our case, a sustained decrease in Hgb levels (81 g/L to 38 g/L) following the use of methylene blue (50 mg) was detected before a clear diagnosis of G6PD deficiency, which is consistent with previous studies.^[3]^ When the patient was confirmed to have favism, methylene blue was discontinued and subsequent treatment with vitamin C and methylprednisolone was administered until complete recovery.

However, co-occurrence of hemolytic anemia and methemoglobinemia in elderly patients with G6PD deficiency is rare and most commonly reported due to fava bean ingestion.^[3,9,10]^ As observed in our case study, when hemolysis worsened during methylene blue treatment, it was discontinued and the patient received a transfusion of 2 units of washed RBC. Therefore, caution must be exercised when using methylene blue, especially in patients with G6PD deficiency that has not been previously diagnosed, as it may not effectively reduce MetHgb levels and could worsen hemolytic anemia. Patients known to have G6PD deficiency should avoid fava beans (especially fresh raw fava beans) and drugs that induce hemolysis, while, the G6PD gene must be detected in methemoglobinemia patient before the treatment with methylene blue, especially the co-occurrence happened.

## 4. Conclusion

In patients with G6PD deficiency, particularly in the elderly, the co-occurrence of acute hemolytic anemia and methemoglobinemia after fresh raw fava bean ingestion is a rare but potentially serious complication. Care should be taken to evaluate G6PD deficiency as soon as the patient is initially diagnosed with methemoglobinemia. Therefore, it is crucial to identify the causes and co-occurrence promptly to prevent further oxidative hemolysis that may be exacerbated by methylene blue.

## Acknowledgments

The authors would like to express their gratitude to Dr Zhao Ying (First Affiliated Hospital, Zhejiang University School of Medicine) for her valuable assistance in providing feedback and in revising the initial manuscript.

## Author contributions

**Conceptualization:** Binze Chen.

**Data curation:** Binze Chen.

**Formal analysis:** Binze Chen.

**Supervision:** Yanxia Han.

**Writing – original draft:** Binze Chen.

**Writing – review & editing:** Yanxia Han.

## References

[1] Luzzatto L Ally M Notaro R. Glucose-6-phosphate dehydrogenase deficiency. Blood. 2020;136:1225–40. 32702756 10.1182/blood.2019000944

[2] Beretta A Manuelli M Cena H. Favism: clinical features at different ages. Nutrients. 2023;15:343. 36678214 10.3390/nu15020343 PMC9864644

[3] Ata F Javed S Muthanna B. Favism-induced methemoglobinemia in a G6PD deficient male with a subsequent hemolytic cascade, a therapeutic challenge: case report and review of literature. Clin Case Rep. 2021;9:2048–52. 33936638 10.1002/ccr3.3941 PMC8077420

[4] Pes GM Dore MP. Acquired glucose-6-phosphate dehydrogenase deficiency. J Clin Med. 2022;11:6689. 36431166 10.3390/jcm11226689 PMC9695330

[5] Li Z Huang Z Liu Y. Genotypic and phenotypic characterization of glucose-6-phosphate dehydrogenase (G6PD) deficiency in Guangzhou, China. Hum Genomics. 2023;17:26. 36949502 10.1186/s40246-023-00473-9 PMC10035184

[6] Malakah MA Baghlaf BA Alsulami SE. Co-occurring hemolysis and methemoglobinemia after COVID-19 infection in patient with G6PD deficiency. Cureus. 2023;15:e35020. 36938163 10.7759/cureus.35020 PMC10022702

[7] Arun VJ Deorukhkar A Rafi AM. Successful management of methemoglobinemia and G6PD deficiency in a patient posted for surgical excision of branchial cyst. Asian J Transfus Sci. 2022;16:128–31. 36199403 10.4103/ajts.ajts_152_20 PMC9528542

[8] Diegues A Simões P Ceriz T Lopes AR Tomé E. Favism: a case report. Cureus. 2022;14:e23269. 35449616 10.7759/cureus.23269 PMC9013287

[9] Schuurman M van Waardenburg D Da Costa J Niemarkt H Leroy P. Severe hemolysis and methemoglobinemia following fava beans ingestion in glucose-6-phosphatase dehydrogenase deficiency: case report and literature review. Eur J Pediatr. 2009;168:779–82. 19263080 10.1007/s00431-009-0952-x

[10] Al-Dubai H Al-Mashdali A Hailan Y. Acute hemolysis and methemoglobinemia secondary to fava beans ingestion in a patient with G6PD deficiency: a case report of a rare co-occurrence. Medicine (Baltimore). 2021;100:e27904. 34964759 10.1097/MD.0000000000027904 PMC8615397

[11] Ata F Muthanna B Javed S Uddin M Yassin M. Favism induced methemoglobinemia in G6DP deficient patients: case series and review of literature. Blood. 2020;136:11–2.32276273

[12] Pushparajah Mak RS Liebelt EL. Methylene blue: an antidote for methemoglobinemia and beyond. Pediatr Emerg Care. 2021;37:474–7. 34463662 10.1097/PEC.0000000000002526

[13] Tang ASO Wong QY Yeo ST. Challenges in managing a lepromatous leprosy patient complicated with melioidosis infection, dapsone-induced methemoglobinemia, hemolytic anemia, and lepra reaction. Am J Case Rep. 2021;22:e931655. 34038399 10.12659/AJCR.931655 PMC8165492

[14] Tiwari AK Aggarwal G Gupta VK Arora D Pabbi SM Rawat G. Automated-red cell exchange for methaemoglobinaemia in a G6PD-deficient patient. Natl Med J India. 2020;33:149–51. 33904418 10.4103/0970-258X.314007

